# Lifestyle Intervention Framework for Obese Patients with Non-alcoholic Fatty Liver Disease – a Tool for Health Professionals in Resource Constraint Settings

**DOI:** 10.7759/cureus.5999

**Published:** 2019-10-25

**Authors:** Charu Arora, Anita Malhotra, Piyush Ranjan, Naval K Vikram, Shalimar ., Namrata Singh, Sada Nand Dwivedi

**Affiliations:** 1 Food and Nutrition, University of Delhi, New Delhi, IND; 2 Food and Nutrition, Lakshmibai College, University of Delhi, New Delhi, IND; 3 Medicine, All India Institute of Medical Sciences, New Delhi, IND; 4 Gastroenterology and Human Nutrition, All India Institute of Medical Sciences, New Delhi, IND; 5 Biostatistics, All India Institute of Medical Sciences, New Delhi, IND

**Keywords:** obesity, lifestyle, intervention, framework, dietitian, non-alcoholic fatty liver disease (nafld)

## Abstract

Purpose

Non-alcoholic fatty liver disease (NAFLD), is recognized as a health care burden worldwide. Lifestyle modification remains the first line of treatment. However, the real challenge is ensuring the patient's adherence to lifestyle modification measures, especially in hospitals with resource-limited settings.

Methods

We developed a six-month-long, dietitian-led, hospital-based, lifestyle intervention framework for obese NAFLD patients and evaluated its content. Literature review, interviews, and discussions with 10 health experts (general physicians, dietitians/nutritionists, gastroenterologists, and a clinical psychologist) and 45 NAFLD patients (35 in Phase I and 10 in Phase II) in a tertiary hospital of India were carried out.

Results

The lifestyle intervention framework has unique features, such as an intensive nature to ensure adherence, a comprehensive educational format with clear guidelines, the customization of a prescription as per individual patient requirements, and a holistic approach to inculcate self-monitoring and behavioral change in NAFLD patients.

Conclusion

Health professionals worldwide can use this lifestyle intervention framework to develop counseling interventions for better adherence among obese NAFLD patients.

## Introduction

Non-alcoholic fatty liver disease (NAFLD) is a rapidly emerging global health concern. Its prevalence is rising remarkably, in parallel to that of obesity, especially in developing countries such as India [1]. This silent liver condition, if left ignored, may progress to dreadful consequences, such as liver cirrhosis and hepatocellular carcinoma [2]. The potential role of lifestyle modification in reversing NAFLD has been well-documented. Not only weight loss, but lifestyle interventions also benefit NAFLD patients in terms of reduction in liver enzymes, reduction in liver fat, and improvement in liver histology [3].

Successful management of NAFLD is highly influenced by the patient’s compliance to the lifestyle modification prescription [4]. However, 50% of NAFLD patients are non-compliant to dietary and lifestyle change advice due to the asymptomatic nature of the disease [5]. A few elaborate lifestyle interventions have been successfully tried in NAFLD patients in developed nations, such as the United States (US) and Italy. On the other hand, the standard care of NAFLD patients in hospitals of developing countries like India involves a quick suggestion of lifestyle modification and prescription of medications for comorbidities by the physician, which is probably not enough to bring out clinically significant results in these patients [2].

Internists and primary care physicians are the first points of contact for most of the NAFLD patients. These patients are referred to dietitians for dietary advice and to gastroenterologists for the various complications of NAFLD. Adherence to lifestyle-related advice is poor and researchers from different parts of the world are now focusing on identifying ways to ensure adherence to lifestyle modification advice by the patients to manage this rising trend of NAFLD. The lifestyle interventions used in developed nations cannot be generalized for patients across developing countries because of different socioeconomic dynamics, different eating habits, cultural and lifestyle differences, differences in infrastructure and resource availability, behavioral differences affecting adherence, etc.

The challenge lies in developing well-defined, feasible, protocolized counseling interventions, which can also be used in the hospitals of developing nations with constrained resources, to bring about clinically significant and positive patient outcomes in obese NAFLD cases. Only a couple of studies with small sample sizes and other research design-based limitations have used a lifestyle modification intervention on NAFLD patients and they provide limited information about how these interventions were developed and applied on the participants [6-7].

Our study explains in detail the systematic process of development and content evaluation of a framework for lifestyle intervention focusing on the treatment needs of Indian NAFLD patients in a government hospital in India. This will assist metabolic physicians and dietitians, who are the first point of contact for NAFLD patients, to better manage their NAFLD patients by ensuring an emphasis on adherence to the lifestyle change prescription.

## Materials and methods

Ethical approval and informed consent

Ethical clearance for the study was approved by the Institutional Ethics Committee of All India Institute of Medical Sciences, New Delhi (Ref. #IEC-434/04.08.2017). The informed consent of all participants was obtained. The study was carried out from January 2018 to July 2018 in two phases - the development of a framework for the intensive lifestyle counseling intervention and its subsequent content evaluation.

This intervention framework development was carried out at one of the biggest government-funded, tertiary care and research hospitals in India. Patients from all socioeconomic, educational, and cultural backgrounds visit this hospital for treatment. Forty-five obese English/Hindi-speaking adults diagnosed with NAFLD attending the Obesity and Metabolic Clinic at this hospital formed the study population. Ten health experts from different fields, such as medicine, gastroenterology and human nutrition, dietetics, and psychology were involved in the study.

Phase I - development of a lifestyle intervention framework

The development of the framework for lifestyle intervention involved literature review and in-depth discussions with subject experts and NAFLD patients.

Literature Review

A comprehensive literature review was carried out to look for education material and pre-existing lifestyle interventions for NAFLD and other lifestyle-related diseases. “MeSH” terms such as “obesity,” “lifestyle intervention,” and “dietary intervention” were used in PubMed and other medical search engines such as Google Scholar and Science Direct to look for studies done over the past 10 years (2008 - 2018). A total of 21 studies were selected based on year of publication, age group of subjects, nature of the intervention, outcome measures used in the study, etc. Selected papers were then studied in detail.

Discussions with Experts and NAFLD Patients

A literature review was followed by detailed discussions with health experts in the field of NAFLD. Three sessions over a time span of six-months were conducted with 10 professionals, including four experts from the field of nutrition and dietetics, three general physicians from the Department of Medicine, two senior gastroenterologists from the Department of Gastroenterology and Human Nutrition, and one associate professor from the Department of Clinical Psychology. The discussions aimed at eliciting from the experts the barriers they face while treating and/or counseling a NAFLD and/or obese patient. Thereafter, 35 obese patients already undergoing treatment of NAFLD at the Obesity and Metabolic Clinic in the hospital were interviewed to collect data on the barriers and facilitators that they face while following the prescribed treatment. We tried to understand the patients’ expectations from the health care professionals during a lifestyle modification prescription.

Based on experts’ suggestions and need assessment of the patients, a framework for a six-month-long, intensive, dietitian-led, lifestyle counseling intervention was developed for NAFLD patients. The goals of the lifestyle intervention were 1) 5% - 10% weight loss, 2) improvement in anthropometric parameters, 3) normalization of liver enzymes, and 4) improvement of CAP score in Fibroscan® (Echosens™, Waltham, MA) within six months.

Identification of Content Based on Literature Review, Expert Advice, and Patients’ Need Assessment

Relevant areas for education and counseling of NAFLD patients were identified after the above steps. After final selection, the content was categorized into different sessions, such as the introduction to NAFLD and its management, the role of dietary behavior in NAFLD, the importance of weight control, the role of exercise in NAFLD and myths about weight loss, and staying motivated in the long run. The content was used to develop counseling tools, such as Microsoft® PowerPoint (Microsoft® Corp., Redmond, WA) presentations, handouts, recipe booklets, exercise demonstrations, food diary, selection of available smartphone applications, and a feedback form for intensive lifestyle intervention. These tools were used during different sessions of the intervention.

Phase II - content evaluation of the lifestyle intervention framework

After developing the framework for intervention, its content was evaluated. The analysis of scores and comments given by experts and patients was used to revise the intervention.

Experts (general physicians, dietitians/nutritionists, gastroenterologists, clinical psychologist) who provided inputs for the content development of the framework were involved in the content evaluation of the proposed framework and tools developed for the intervention. The intervention tools developed were pilot tested on 10 new NAFLD patients (who did not participate in Phase I of the study).

Evaluation of the Lifestyle Intervention Framework and Tools by Health Experts

The proposed framework for dietary and physical activity intervention was evaluated by 10 health experts from the fields of gastroenterology, human nutrition, dietetics, medicine, and clinical psychology. The scoring was done on a scale of 1 to 10 over three parameters - content, the layout of the framework, and tools developed for the intervention. 

Pilot Testing of Intervention Tools on a Small Sample of NAFLD Patients

The intervention aids of the framework were then pilot-tested on 10 NAFLD patients to observe the patients’ acceptance and comprehension of the education imparted through the tools. The patients marked the counseling intervention material on a scale of 1 to 10 based on parameters, such as ease of understanding and layout.

Revision of Content as Per the Suggestions Received

In the final step, the framework was revised on the basis of comments received from experts and patients.

## Results

The final framework for the intervention after incorporating the suggestions/observations of experts and NAFLD patients in the evaluation phase is shown in Table 1. The framework lists nine sessions of which five are face-to-face and four are telephonic sessions. The theme, timing, duration, and tools to be employed, and specific objectives, along with intervention guidelines, are given in detail in Table 1. 

**Table 1:** Objectives, Duration, and Content of the Intervention Framework (Session-wise) *Microsoft® Corp., Redmond, WA NAFLD: non-alcoholic fatty liver disease

Session 1: (Face-to-face)	
a) Theme: Introduction to fatty liver disease and its management	a) OBJECTIVES: To form a rapport with the patient and accompanying family member, to introduce the disease and its management, and to highlight the goal of 5% - 10% bodyweight reduction in six months.
b) Timing: On the day of enrollment	
c) Duration: 60 minutes	b) INTERVENTION: Rapport formation with the patient and family members, introduce the role of lifestyle management in NAFLD, explain the meaning of the deranged metabolic parameter, set small and achievable goals for the patient, and encourage buying of a weighing scale; Customized diet pattern, based on the principles of a balanced diet with a one-day sample diet plan (based on economic, cultural, regional preferences and associated symptoms and comorbidities with NAFLD); Introduce the concept of “Sitting less for a healthy liver” and the benefits of regular walking in NAFLD; Teach to write a food diary; Exchange phone numbers
d) Tools employed: Microsoft ® Powerpoint* presentation, a sample of a food diary, customized diet chart	
Session 2: (Telephonic)	
a) Session theme: Reinforcement of prescription	a) OBJECTIVES: To strengthen the rapport and emphasize on the prescribed diet.
b) Timing: within 15 days from the first session;	b) INTERVENTION: Reinforce the prescription, explaining the importance of a balanced yet calorie-controlled diet; Emphasize the concept of small and frequent meals; Motivate to maintain a food diary; Clarify doubts, if any
c) Duration: 10 - 15 minutes	
d) The tool employed: Checklist	
Session 3 (Telephonic)	
a) Session theme: Monitoring of compliance and progress	a) OBJECTIVES: To monitor the progress of the patient in terms of dietary and physical activity changes, as well as weight loss; To motivate the patient to adhere to the prescription. b)
b) Timing: At the completion of one month	
c) Duration: 10 - 15 minutes	b) INTERVENTION: Inquire whether the recommended changes have been made in the daily diet and physical activity; Inquire about the weight loss; Explain the progress to the patient and motivate for adherence and further improvement (if required); Clarify doubts, if any
d) The tool employed: Checklist	
Session 4 (Face-to-face)	
a) Session theme: Weight management and meal planning	a) OBJECTIVES: To empower the patient with the basic knowledge of meal planning for healthy eating; To emphasize the role of exercise in weight loss and improvement of fatty liver disease.
b) Timing: At the completion of two months	
c) Duration: 30 - 45 minutes	b) INTERVENTION: Check the food diary to understand the routine of the patient; Session on how to plan your meal and recommended food group intake; Role of exercise in weight management and setting your exercise targets
d) The tool employed: Microsoft® PowerPoint presentation	
Session 5 (Face-to-face)	
a) Session theme: Progress report and way ahead	a) OBJECTIVES: To reset a new diet and exercise goal for the patient as per their progress in the preceding three months; To explain the journey of weight loss and associated barriers to the patient.
b) Timing of the session: At the completion of three months	
c) Duration: 30 minutes	b) INTERVENTION: Check the food diary to understand the routine of the patient; Give a new customized diet plan based on the current need of the patient (weight loss or maintenance); Explain possible reasons to the patient, in cases where no progress is made; Explain the plateau effect and measures to overcome it; Set new weight loss and exercise targets for the patients, if required; Give a recipe booklet on low calorie snack
d) Tools employed: Handouts, recipe booklet, and diet chart	
Session 6 (Telephonic)	
a) Session theme: Motivation and reminder	a) OBJECTIVES: To motivate the patient to continue with the prescribed lifestyle prescription; To make the patient feel positive.
b) Timing of session: At the completion of three months, 15 days	
c) Duration: 10 - 15 minutes	b) INTERVENTION: Reminder call to check progress and keep motivation high.
d) The tool employed: Checklist	
Session 7 (Telephonic)	
a) Session theme: Compliance check and motivation	a) OBJECTIVES: To motivate the patient to continue with the prescribed lifestyle prescription; To make the patient feel that he is being constantly monitored.
b) Timing: At the completion of four months	
c) Duration: 10 - 15 minutes	b) INTERVENTION: Reiterate the role of weight loss in the improvement of liver function; Motivate the patient to maintain a positive mind frame regarding a permanent lifestyle change
d) Tools employed: None	
Session 8 (Face-to-face)	
a) Session theme: Managing your eating behavior	a) OBJECTIVE: To empower the patient to make informed food choices that are healthy.
b) Timing: At the completion of five months	b) INTERVENTION: Education session on how to read nutrition labels? Show some sample labels and their interpretation; How to eat healthy while eating out; Identifying cravings, reaction to cravings, and how to get back on track; How to control social influences to not disturb your diet; Simple sugar and fat amounts in some snacks and beverages
c) Duration: 30 minutes	
d) Tools employed: Food labels, handouts	
Session 9 (Face-to-face)	
a) Session theme: Final assessment and way ahead with self-management	a) OBJECTIVES: To analyze the progress made by the patient during the 6-month self-management in terms of metabolic parameters To enhance motivation to maintain behavior change by recognizing personal successes so far.
b) Timing: At the completion of six months	
c) Duration: 30 minutes	b) INTERVENTION: Final progress assessment of the patient; Helping the patient develop a healthy eating and exercise pattern for the future; Busting some myths about weight loss; How to stay motivated in the long run?
d) The tool employed: Handouts	

## Discussion

In this study, we have developed a framework for lifestyle intervention for NAFLD patients. This intervention counseling framework is a combination of dietary, exercise, and behavior strategies, combined in a manner so that they do not overwhelm the participant nor does it create too much burden on the health professional/dietitian working with these patients in a resource-constrained setting.

It is a goal-based behavioral intervention framework that also provides the required flexibility to customize the methods suitable for each individual to achieve these goals. It involves individualized counseling, keeping in mind the education, previous medical history, and family background of the patient. To ensure compliance with the lifestyle change prescription, an intensive approach to lifestyle modification has been incorporated in this intervention framework. The component of time to timed phone calls and messages to the patients by a registered dietitian ensures that they do not lose the motivation to follow a healthy lifestyle. The intervention framework is comprehensive and focuses on nutrition education and health promotion through self-monitoring of diet, physical activity, and weight by the patient (Table 1).

A few international trials, such as the Look AHEAD (Action for Health in Diabetes) and Diabetes Prevention Program (DPP) studies, have demonstrated the benefits of lifestyle intervention in metabolic diseases, such as diabetes and obesity [8-10]. Although the principles of lifestyle management in diabetes, obesity, and NAFLD are somewhat similar, no independent large scale lifestyle modification trial has been conducted in NAFLD patients across the globe. A fatty liver ancillary study was carried out at one of the 16 Look AHEAD clinical sites with 244 participants [11]. The participants were encouraged to lose at least 10% of initial weight at 12 months through a combination of moderate caloric restriction and increased physical activity. During the first six months, participants attended weekly meetings (one individual and three group sessions per month). During the remaining months, the participants attended monthly individual and group sessions. This study reported that intensive lifestyle intervention produced 8% weight loss along with a significant improvement in hepatic steatosis and a substantially lower incidence of NAFLD compared to the control group [11]. Another similar study was performed in the year 2009 by Promrat et al. to examine the effects of lifestyle intervention using a combination of diet, exercise, and behavior modification with a goal of 7% to 10% weight reduction on clinical parameters of nonalcoholic steatohepatitis (NASH) [12]. They also used an intensive lifestyle intervention designed on the basis of the Look AHEAD and DPP trials. This 48-week-long intervention concluded that weight reduction done through intensive lifestyle intervention leads to improvements in liver histology in non-alcoholic steatohepatitis. A randomized controlled trial from Hong Kong has successfully demonstrated the use of a dietitian-led lifestyle modification intervention to reduce liver fat in NAFLD patients [13]. However, this intervention was of 12 months in duration and was implemented in a community-based setting, unlike our intervention framework, which was developed for use in a hospital setting. The intervention tools to be used in various sessions were developed both in English and Hindi to ensure ease of understanding by non-English-speaking patients attending our hospital. The intervention uses diverse tools and techniques, such as discussions, handouts, Microsoft® PowerPoint presentations (Microsoft® Corp., Redmond, WA), food diary, demonstrations, etc. to engage the patient in the lifestyle modification journey. Our intervention framework can also be compared to a study conducted on Korean NAFLD patients, which employed a mix of telephonic and face-to-face nutrition education sessions for the remission of NAFLD [14]. However, nutritional education was imparted using only a booklet and handout in this study. The intervention framework designed by us combines the aspect of diet, as well as physical activity, along with a behavioural modification to treat NAFLD. Many interventions done on NAFLD patients include either the diet [15-16] or exercise component [17-18] only. Also, many studies done on NAFLD patients are based on specific diets, such as the Mediterranean diet [19] and the ketogenic diet [15, 20]. Our intervention framework is based on the balanced diet approach with individualized calorie reduction goals (500 - 1,000 kcal/day) targeted towards gradual weight loss, similar to a six-month exclusive nutrition intervention used by Elias et al. on NAFLD patients [21]. Another highlight of our study is that the intervention framework has been designed in consultation with many health experts from diverse fields, such as gastroenterology, nutrition, clinical psychology, and medicine. Efforts have also been made to ensure that most of the barriers reported by the physicians and dietitians during the lifestyle counseling of NAFLD patients are addressed through this intervention.

The distinct characteristics, which make our intervention framework unique, are its intensive nature, educational format, individual-based customization, and holistic approach to inculcate self-monitoring and behavioral change in NAFLD patients and the adaptive nature to fit in the requirements of resource-constrained settings. However, our intervention framework has its own limitations. Though its content has been validated by experts, its efficacy against the standard lifestyle change prescription has yet to be assessed as part of a trial. Also, assessment of knowledge about the disease and subsequent modification in the attitude and practice is extremely effective in treating lifestyle diseases. Knowledge, attitude, and practice (KAP) is an important component of the knowledge-attitude-behavior model, which proposes that accumulated knowledge about a health aspect initiates change in attitude and results in gradual behaviour change. It is equally important to regularly monitor the adherence of the patient to the behaviour change and understand the determinants of non-adherence. Medical practitioners and other healthcare providers can use validated KAP and adherence questionnaires [22-23] in lifestyle-related diseases, alongside this educational intervention, to give a more holistic lifestyle counseling experience to the patient. 

With minor customizations based on the economic feasibility and availability of resources, healthcare professionals involved in the management of NAFLD all over the world can make use of this intensive dietary intervention framework to suit the needs of their patients.

## Conclusions

This study has developed a holistic, scientifically designed, education-based, intensive, personalized, expert-validated, and goal-oriented framework for intensive dietary counseling of obese NAFLD patients in resource-constrained settings. This intervention framework may prove to be instrumental for dietitians and health professionals in India, as well as other parts of the world, working in the field of NAFLD to develop a systematic way of lifestyle counseling and thereby improve adherence to prescription among their patients.
